# Data Mining of Atherosclerotic Plaque Transcriptomes Predicts STAT1-Dependent Inflammatory Signal Integration in Vascular Disease

**DOI:** 10.3390/ijms150814313

**Published:** 2014-08-18

**Authors:** Krzysztof Sikorski, Joanna Wesoly, Hans A. R. Bluyssen

**Affiliations:** 1 Department of Human Molecular Genetics, Adam Mickiewicz University in Poznan, Poznan 61-614, Poland; E-Mail: h.bluyss@amu.edu.pl; 2 Laboratory of High-Throughput Technologies, Institute of Molecular Biology and Biotechnology, Adam Mickiewicz University, Umultowska 89, Poznan 61-614, Poland; E-Mail: j.wesoly@amu.edu.pl

**Keywords:** atherosclerosis, JAK-STAT signalling, IFNγ, TLR4, diagnostic markers

## Abstract

Atherosclerotic plaque development involves multiple extra- and intra-cellular signals engaging cells from the immune system and from the vasculature. Pro-inflammatory pathways activated by interferon gamma (IFNγ) and toll-like receptor 4 (TLR4) ligands are profoundly involved in plaque formation and have been shown to involve cross-talk in all atheroma-interacting cell types leading to increased activation of signal transducer and activator of transcription-1 (STAT1) and elevated expression of pro-inflammatory mediators. Here we demonstrate that in Gene Expression Omnibus repository (GEO) deposited microarray datasets, obtained from human coronary and carotid atherosclerotic plaques, a significant increase in expression of pro-inflammatory and immunomodulatory genes can be detected. Moreover, increased expression of multiple chemokines, adhesion molecules and matrix-remodeling molecules was commonly detected in both plaque types and correlated with the presence of putative STAT1 binding sites in their promoters, suggesting strong involvement of STAT1 in plaque development. We also provide evidence to suggest that STAT1-nuclear factor kappa-light-chain-enhancer of activated B cells (NFκB) or STAT1-interferon-regulated factor (IRF) regulatory modules are over-represented in the promoters of these inflammatory genes, which points to a possible contribution of IFNγ and TLR4 cross-talk in the process of atherogenesis. Finally, a subset of these genes encodes for secreted proteins that could serve as a basis of a non-invasive diagnostic assay. The results of our *in silico* analysis *in vitro* provide potential evidence that STAT1-dependent IFNγ-TLR4 cross-talk plays a crucial role in coronary and carotid artery plaque development and identifies a STAT1-dependent gene signature that could represent a novel diagnostic tool to monitor and diagnose plaque progression in human atherosclerosis.

## 1. Introduction

Developing atherosclerotic plaque integrates multiple extra- and intracellular signals recognized by immune as well as vascular cells [1,2,3]. Activation of multiple signaling pathways in all cell types present in the atheroma leads to cross-talk at the level of cell-cell interaction but also at that of transcription factor activation. For instance, endothelial and smooth muscle cells respond to damage and pathogen associated molecular patterns, which activate Toll-like receptors (e.g., TLR4) [4]. The same ligands activate T_H_1 lymphocytes to release pro-inflammatory interferon (IFN)γ, which also acts on vascular cells [5]. Consequently, cell activation leads to release of inflammatory cytokines and chemokines and presentation of adhesion molecules, allowing leukocyte infiltration of the vessel wall and plaque progression [6]. It has been shown in many studies that IFNγ plays a key role in atherogenesis [7,8]. IFNγ activates an inflammatory transcriptional program through the canonical janus-kinase-signal transducer and activator of transcription (JAK)-STAT signaling pathway. This involves early activation of STAT1 (Signal Transducer and Activator of Transcription-1) and, at a later stage, of IRFs (Interferon Regulatory Factors), to generate a prolonged interferon response [9]. The immediate IFNγ response involves binding of STAT1 dimers to the Gamma Activated Sequence (GAS) element in the promoters of genes such as interferon-regulated factor 1 (IRF1), guanylate binding proteins (GBP) and intercellular adhesion molecule 1 (ICAM1). In contrast, the IRF-mediated response to IFNγ utilizes the Interferon Stimulated Response Element (ISRE) to induce expression of chemokine genes such as chemokine (C-C motif) ligand 5 CCL5 [10] or C-X-C motif chemokine 10 (CXCL10) [11].

Similar to IFNγ, TLR4 expression was detected in human and mouse atherosclerotic plaques. Moreover, circulating monocytes from acute coronary syndrome and coronary arteriosclerotic patients exhibit elevated TLR4 expression [12]. Mice deficient in TLR4 show reduced atherosclerosis proving that Toll-like receptor-dependent signaling participates in disease development [13]. Signaling of TLR4 involves two major pathways: 1. MyD88 (Myeloid Differentiation factor 88)-dependent NFκB (Nuclear Factor kappa B) activation and TRAM (TLR4 adaptor molecule)-dependent IRF3 activation. NFκB induces expression of many inflammatory genes, such as inducible nitric oxide synthase (iNOS), tumor necrosis factor alpha (TNFα) and interleukin 6 (IL6). IRF3, on the other hand, induces production of IFNβ, which in an auto- and paracrine manner activates STAT1 [14]. Thus, IFNγ and TLR4 signaling pathways utilize common transcription factors, including STAT1 and IRFs.

Over the years, cross-talk has been shown to exist between IFNγ and lipopolysaccharide (LPS) in the different cell types building the atherosclerotic plaque, resulting in increased expression of inflammatory mediators [15]. This cross-talk encompasses a complex, multi-layered mechanism relying on increased activation of STAT1 as well as on interactions of STAT1 with other transcription factors (*i.e.*, IRF1 and NFκB), resulting in increased expression of genes such as CXCL10 and ICAM1 [11,16,17]. The interactions can be either direct at the protein level or indirect at the level of promoter binding sites. The latter requires specific regulatory modules containing STAT1 binding elements and NFκB or IRF binding sites in close proximity. Similar cross-talk phenomena have been proven to exist for other cytokine combinations (e.g., TNFα and IFNγ, and IL1β with IFNγ) and to particularly contribute to inflammatory gene expression. For instance, IFNγ and TNFα synergistically induced CXCL9 [18], ICAM1 [19] and iNOS [20], which depended on GAS and NFκB elements in their promoters and involved interaction of bound STAT1 and NFκB factors with CREB-binding protein and enhanced recruitment of RNA polymerase II [18]. Alternatively, the human IDO1 gene and murine Tap1 and Lmp2 genes were shown to possess combined ISRE and gamma-interferon-activated sites (GAS) elements in their promoters, both being necessary for maximum induction by IFNγ [21,22]. A third possible mechanism was revealed in the regulation of vascular cell adhesion molecule 1 (VCAM1) [19] and CCL19 [23] expression, where a combination of interferon-stimulated response element (ISRE) and NFκB elements appeared to be responsible for optimal transcription. In case of CXCL10, an even more complex mechanism seems to be involved, since synergistic induction by IFNγ and TNFα relied on ISRE and NFκB elements [11], whereas IFNγ-TLR4 cross-talk was suggested to depend on STAT1 [24].

Until now, the existence of these different cross-talk mechanisms has been revealed predominantly by *in vitro* experiments conducted in the individual cell types involved in atherosclerotic plaque formation. To date there is limited information available on the role of IFNγ and TLR4 signaling cross-talk in the regulation of pathophysiological processes underlying atheroma development. Here, by applying an *in silico* approach, we analyzed gene expression profiles in combination with gene ontology (GO) classification and promoter analysis of human coronary and carotid lesions (extracted from GEO: GSE40231 [25] and GSE21545 [26]) for potential evidence that STAT1-dependent inflammatory signal integration may be involved in plaque development. Indeed, our analysis highly suggests that STAT1-NFκB and STAT1-IRF regulatory modules are over-represented in promoters of inflammatory genes up-regulated in human coronary and carotid plaques and points to a possible involvement of IFNγ and TLR4 cross-talk.

Moreover, based on GO classification of these up-regulated genes, we detected high similarity in molecular processes and cellular interactions underlying plaque development in both vessel types, predicting overlap in pathophysiology. Finally, this comparative gene expression analysis revealed the presence of a common subset of inflammatory chemokine, cytokine and matrix remodeling genes, encoding for secreted proteins. These could serve as a basis of a non-invasive diagnostic assay for early detection and monitoring of the atherosclerotic process.

## 2. Results

### 2.1. STAT1 Target Genes Are Profoundly Present in Coronary Plaques

First, we analyzed a microarray dataset obtained from human coronary plaques. The dataset is available in the GEO NCBI database (acc. no. GSE40231 [25]). We evaluated 254 genes up-regulated at least two-fold as compared to the control, healthy arterial tissue. The full list of differentially regulated genes is available in supplementary data. When we examined the top 20 up-regulated genes, we immediately recognized a distinctive inflammatory transcriptional program, including proteins involved in cell-cell adhesion and trans-endothelial migration (DSC3, CDH2), cell-matrix adhesion (HAPLN1) and cytokine signaling (TNFRSF11, CYTL1, CARTPT, IL13RA2). Secreted phosphoprotein 1 (SPP1) was shown to be up-regulated in calcified lesions [27] and also to up-regulate IFNγ [28], and IL12 and SCG2 expression is stimulated by oxidized low-density lipoprotein (oxLDL) in macrophages and dendritic cells [29]. We then inspected the whole list in a more global way by conducting functional analysis and gene ontology (GO) enrichment studies, which showed significant over-representation of GO terms related to: adhesion, migration, immune response, *etc.* Table 1 shows the 20 terms with the strongest enrichment, a full list of these terms is available in the supplementary data file. These processes are hallmarks of atherosclerotic plaque formation. We then analyzed the presence of potential STAT1 binding sites in the promoters of the up-regulated genes and found that GAS and ISRE elements were both over-represented with z-scores of 5.14 and 3.63, respectively (Table 2). Among 62 genes possessing one of these elements we found multiple chemokines (CCL2, CCL5, CCL19, CXCL10, CXCL9), cytokines (VEGFC), adhesion molecules (integrins), and proteins involved in matrix remodeling (MMP19) (also consult the supplementary data). 

**Table 1:** Twenty most enriched gene ontology (GO) terms in the coronary dataset.

GO Term	GO ID	log10 *p*-Value
response to external stimulus	GO:0009605	−12.1409
biological adhesion	GO:0022610	−11.2716
single-multicellular organism process	GO:0044707	−11.2716
locomotion	GO:0040011	−11.0788
multicellular organismal process	GO:0032501	−10.5817
single-organism process	GO:0044699	−10.0362
developmental process	GO:0032502	−9.0752
anatomical structure development	GO:0048856	−8.7645
single-organism cellular process	GO:0044763	−8.4034
response to stimulus	GO:0050896	−8.2916
signalling	GO:0023052	−5.8633
single organism signalling	GO:0044700	−5.8633
localization of cell	GO:0051674	−5.4306
response to chemical stimulus	GO:0042221	−4.3116
immune system process	GO:0002376	−3.6253
regulation of biological quality	GO:0065008	−3.4145
behaviour	GO:0007610	−3.2549
response to abiotic stimulus	GO:0009628	−2.6289
rhythmic process	GO:0048511	−2.3675

**Table 2:** Over-representation of regulatory elements in promoters of genes from all datasets. nuclear factor kappa-light-chain-enhancer of activated B cells (NFκB) and interferon-regulated factor (IRF) refer to signal transducer and activator of transcription-1 (STAT1)-NFκB and STAT1-IRF modules. The numbers shown are z-scores.

	STAT1		NFκB	IRF
	GAS	ISRE		
coronary	5.14	3.63	5.05	3.66
carotid	4.92	6.39	5.23	6.29
common	3.24	2.06	2.87	4.86

As explained in the introduction, a distinct feature allowing for signaling cross-talk between IFNγ and TLR4 is the presence of regulatory modules in promoters of synergistically induced genes. We found that STAT1-NFκB and STAT1-IRF modules were over-represented with z-scores of 5.05 and 3.66, respectively (Table 2). Genes with putative STAT1-IRF modules again reflected mechanisms underlying plaque formation: cell adhesion (NCAM1, VCAM1, THBS1) and migration (CCL19, CCL2, CCL4 and CCRL1), matrix remodeling and calcification (ADAMTS9, SPP1, MMP19) and inflammatory signaling (IL7R, IL13RA2, TLR4, TNFRSF10B, TNFRSF11B). Putative STAT1-NFκB modules were present in a similar set of genes (also consult the supplementary data file).

### 2.2. Carotid Plaques Share with Coronary Plaques Similar Pro-Inflammatory Gene Expression Patterns Governed by STAT1

In addition to the coronary dataset (GSE40231) we analyzed microarrays from dataset GSE21545 [26], containing carotid plaque samples. A full list of genes with significantly changed expression is available in the supplementary data. We analyzed 845 two-fold up-regulated genes. Similar to coronary plaques we found that the top 20 genes represented inflammatory response and pathophysiology of plaque formation: matrix remodeling and calcification (SPP1, MMP12, MMP9, ACP5), markers of macrophage activation (CHI3L) disturbed composition of lipoproteins (APOC1, PLA2G7), molecules involved in leukocyte adhesion and transmigration (S100A8, S100A9), cell apoptosis (BCL2A1) and antigen processing (IFI30). We took a similar approach as in the case of coronary plaque analysis and looked at global functional changes in the dataset by analyzing enrichment in specific GO terms. We found that genes up-regulated in carotid plaques had functions involved in immune response, adhesion and migration, and response to stress (Table 3).

**Table 3:** Twenty most enriched GO terms in the carotid dataset.

GO Term	GO ID	log10 *p*-Value
immune system process	GO:0002376	−133.4342
immune response	GO:0006955	−120.0516
response to stimulus	GO:0050896	−77.3635
response to biotic stimulus	GO:0009607	−55.3516
response to stress	GO:0006950	−54.1255
response to chemical stimulus	GO:0042221	−53.7212
multi-organism process	GO:0051704	−47.5129
signalling	GO:0023052	−28.9431
single organism signalling	GO:0044700	−28.9431
single-organism cellular process	GO:0044763	−25.752
single-organism process	GO:0044699	−25.5622
biological adhesion	GO:0022610	−23.9788
regulation of biological process	GO:0050789	−23.9706
immune effector process	GO:0002252	−23.7825
biological regulation	GO:0065007	−22.2381
interspecies interaction between organisms	GO:0044419	−22.1319
locomotion	GO:0040011	−22.0991
regulation of molecular function	GO:0065009	−20.7825
regulation of biological quality	GO:0065008	−18.4841

The next step was to assess possible over-representation of putative STAT1 binding sites (Table 2). We found that both GAS and ISRE elements were over-represented with z-scores of 4.92 (GAS) and 6.39 (ISRE). Genes possibly regulated by STAT1 in carotid plaques were mostly chemokines (CCL2, CCL19, CCL5, CCRL2, CCL13). Next, we checked for the presence of STAT1-NFκB and STAT1-IRF regulatory modules and found z-scores of 5.23 (STAT1-NFκB) and 6.29 (STAT1-IRF) (Table 2). STAT1-NFκB and STAT1-IRF modules were present in a similar set of genes: adhesion molecules (ITGAM, PECAM1, VCAM1, ITGB2), chemokines (CCL2, CCL5, CCL18, CCL19, CCL7, CCL8), and matrix remodeling molecules (MMP1, MMP9, MMP12, SPP1).

Together these results highly imply that there are common features between coronary and carotid plaques, possibly regulated by STAT1 and by STAT1-dependent regulatory modules. The similarity of GO terms enriched in both datasets reflects an underlying common biology of the two plaque types.

### 2.3. 72 Genes Are Expressed in both Coronary and Carotid Plaques and Form “Plaque Signature”

**Figure 1:**
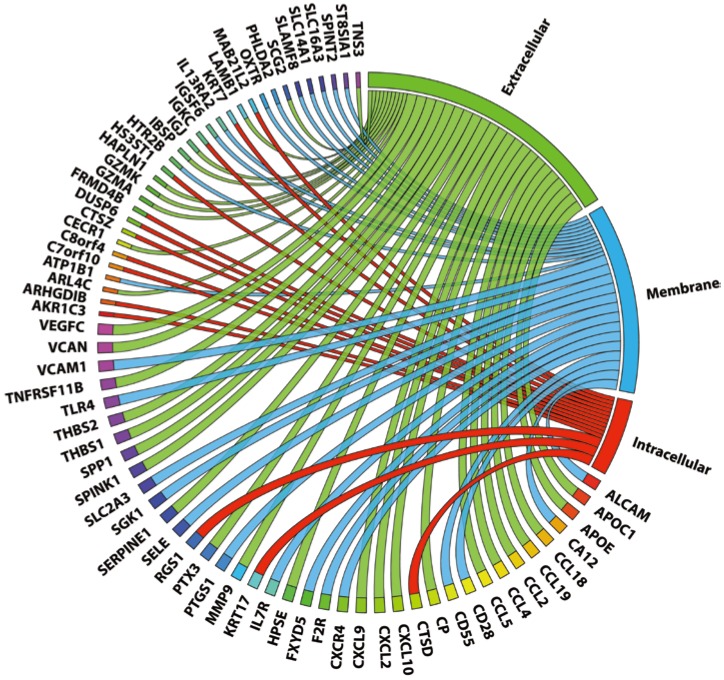
Localization of products of genes expressed in both plaque types. Gene products are organized according to their localization: extracellular (green), cell membrane (blue), and intracellular (red). Wider ribbons represent a known link to atherosclerosis, as determined by literature mining (see Methods).

Finally, we compared genes up-regulated in carotid and coronary datasets and found 72 common genes. Among these genes there are multiple chemokines (CCL18, CCL19, CCL2, CCL4, CCL5, CXCL10, CXCL2, CXCL9), adhesion molecules (HAPLN1, THBS1, THBS2, VCAM1, ALCAM, SELE), matrix remodeling molecules (VCAN, MMP9, LAMB1, HPSE) and also genes with proven involvement in atherosclerosis (APOC1, APOE, CD55, CD69, SCG2, SPP1, TLR4, SLC16A3). When we organized these genes according to localization of their products we found that 30 were secreted and 25 were cell membrane-bound, which makes them good potential biomarkers of the plaque (Figure 1 and Table 4).

**Table 4:** Genes up-regulated in both plaque types, whose products are secreted. The table also shows presence of putative regulatory elements in promoters, if regulation by STAT1 has been proven.

Gene Symbol	Known STAT1 Regulation		ISRE	STAT1-NFκB	STAT1-IRF
APOC1	no		+	−	−
APOE	no		+	−	−
CCL18	no		+	+	+
CCL19	no		+	+	+
CCL2	yes [30,31]		+	+	+
CCL4	yes [32]		+	+	+
CCL5	yes [33]		+	+	+
CP	no		+	−	+
CXCL10	yes [34]		+	+	−
CXCL2	no		−	+	−
CXCL9	yes [34]		+	−	−
GZMA	no		+	−	+
GZMK	no		+	−	+
HAPLN1	no		+	+	+
HPSE	no		−	−	−
IBSP	no		+	−	−
IGJ	no		−	+	+
LAMB1	no		+	+	+
MMP9	yes [35]		−	+	+
PTX3	no		+	−	+
SCG2	no		+	−	+
SERPINE1	yes [36]		+	+	+
SPINK1	no		+	−	+
SPP1	no		+	+	+
THBS1	no		+	−	+
THBS2	no		+	−	−
TNFRSF11B	no		+	+	+
TNS3	no		+	−	−
VCAN	no		+	+	+
VEGFC	yes [37]		+	−	+

### 2.4. Genes Common for Carotid and Coronary Plaques Are Strongly Involved in Plaque Formation Processes and Could Be Regulated by STAT1

Gene ontology classification and enrichment of genes common for both types of plaques showed strong bias towards immune response, cell adhesion and migration, and response to stress (Table 5). 

**Table 5:** Twenty most enriched GO terms obtained from the list of genes common for carotid and coronary plaques.

GO Term	GO ID	log10 *p*-Value
biological adhesion	GO:0022610	−7.8861
immune system process	GO:0002376	−6.2765
locomotion	GO:0040011	−6.2765
single-organism process	GO:0044699	−5.5575
single-organism cellular process	GO:0044763	−5.2832
immune response	GO:0006955	−4.8041
response to stress	GO:0006950	−4.6402
localization of cell	GO:0051674	−4.5607
response to external stimulus	GO:0009605	−4.1475
response to chemical stimulus	GO:0042221	−4.0329
multi-organism process	GO:0051704	−3.2677
multi-multicellular organism process	GO:0044706	−2.7118
regulation of biological quality	GO:0065008	−2.7082
response to biotic stimulus	GO:0009607	−2.6917
localization	GO:0051179	−2.5934
response to stimulus	GO:0050896	−2.121
response to abiotic stimulus	GO:0009628	−2.1199
single-organism developmental process	GO:0044767	−2.0234

Also, binding sites of STAT1 and STAT1-containing regulatory modules were over-represented with z-scores of 3.24 (GAS), 2.06 (ISRE), 2.87 (STAT1-NFκB), 4.86 (STAT1-IRF) (Table 2). All of the 72 common genes had a putative GAS element. STAT1-NFκB module was present in 31 genes from the group, and in eight it was present exclusively. STAT1-IRF module was detected in 45 genes, and 23 did not have the other module. In fact, some of these genes (CCL5, CCL19, CCL4, CXCL10, CXCL2, CXCL9 and MMP9) were actually shown to be co-regulated by STAT1, NFκB and IRFs. Fourty of the 72 genes have a known link to atherosclerosis, as determined by literature mining (Figure 1).

## 3. Discussion

IFNγ and TLR4-mediated signaling pathways, activated in different immunomodulatory and vascular cell types, have been implicated in plaque development and progression [8,13]. Both pathways utilize STAT1 [15] to regulate expression of inflammatory and pro-atherosclerotic genes, such as chemokines (CXCL10) and adhesion molecules (ICAM1) [38]. In our previous research we were able to show *in vitro* that in endothelial cells (ECs) and vascular smooth muscle cells (VSMCs) cross-talk between IFNγ and LPS exists and *in vitro* facilitates STAT1-dependent increase chemokine expression and monocyte to endothelial cells adhesion, a hallmark of early atherosclerosis [17]. A similar STAT1-dependent mechanism was described by others in immune cells [39]. Thus, IFNγ-TLR4 signaling cross-talk importantly controls behavior and interactions of all cells involved in atherosclerotic plaque formation, although this has not been studied in the context of atherosclerotic plaques.

Here we applied an *in silico* approach on deposited in GEO gene expression profiles of coronary and carotid atherosclerotic plaques. Using GO classification, we first conducted functional analysis on 254 up-regulated genes selected from the coronary dataset. This disclosed a statistically significant over-representation of genes involved in cell adhesion, migration, response to external stimulus, and immune response (Table 1). Likewise, functional analysis of 845 up-regulated genes from the carotid dataset revealed strong over-representation of GO terms such as: immune response, adhesion, migration, *etc.* (Table 3), which was similar to our findings in coronary plaques.

These results are in line with the current view of the different processes involved in plaque development and progression [40,41]. First, injured endothelium becomes pro-thrombotic and releases cytokines and chemokines, allowing for leukocyte adhesion and migration into the vessel wall. Then, lymphocytes differentiate into T cells (mostly of the T_H_1 phenotype) and monocytes into macrophages. Subsequent production of inflammatory mediators by these cells activates proliferation and migration of smooth muscle cells facilitated by secretion of matrix-metalloproteinases (by macrophages and smooth muscle cells) [1]. Together with a disturbed composition of plasma lipoproteins, a known risk factor for atherosclerosis [42], all of these processes are reflected in the over-represented GO terms listed in Table 1 and the supplementary data file.

Interestingly, we have observed that 62 out of 254 of the up-regulated genes in coronary plaques possess potential STAT1 binding sites (GAS or ISRE) in their promoters (see Results, Table 2 and supplementary data) highly implicating STAT1 in the development of atherosclerotic plaques. Genes containing either GAS or ISRE elements included chemokines (CCL2, CCL5, CCL19, CXCL10, CXCL9), cytokines (VEGFC), adhesion molecules (integrins), and proteins involved in matrix remodeling (MMP19). In the carotid plaques 208 of 845 up-regulated genes demonstrated presence of putative STAT1 binding sites and involved chemokines (CCL2, CCL19, CCL5, CCRL2, CCL13), chemokine receptors (CX3CR1, CXCR6), cytokines, and cytokine receptors (IL18, IL2RG, IL2RB). These findings are in correlation with results from *in vitro* studies, which showed that many of these genes are indeed STAT1 targets (e.g., CCL5 [16], CXCL10 [43]).

Since our aim was also to provide evidence for the potential role of IFNγ-TLR4 cross-talk in plaque development, we searched for the presence of transcriptional modules of STAT1 and NFκB or STAT1 and IRF binding sites (not more than 50 nucleotides apart). Indeed, we uncovered in coronary plaques that 116 genes contained a STAT1-NFκB module and 150 a STAT1-IRF module, pointing to a potential mechanism of STAT1 dependent co-regulation of gene expression in the cell types present in atherosclerotic plaques.

GO classification of up-regulated genes in coronary plaques with putative STAT1-IRF modules in their promoters reflect mechanisms underlying plaque formation: cell adhesion (NCAM1, VCAM1, THBS1) and migration (CCL19, CCL2, CCL4 and CCRL1), matrix remodeling and calcification (ADAMTS9, SPP1, MMP19), and inflammatory signaling (IL7R, IL13RA2, TLR4, TNFRSF10B, TNFRSF11B). In carotid plaques STAT1-IRF and STAT1-NFκB modules were present in a partially similar set of genes: adhesion molecules (ITGAM, PECAM1, VCAM1, ITGB2), chemokines (CCL2, CCL5, CCL18, CCL19, CCL7, CCL8), and matrix remodeling molecules (MMP1, MMP9, MMP12, SPP1).

**Figure 2:**
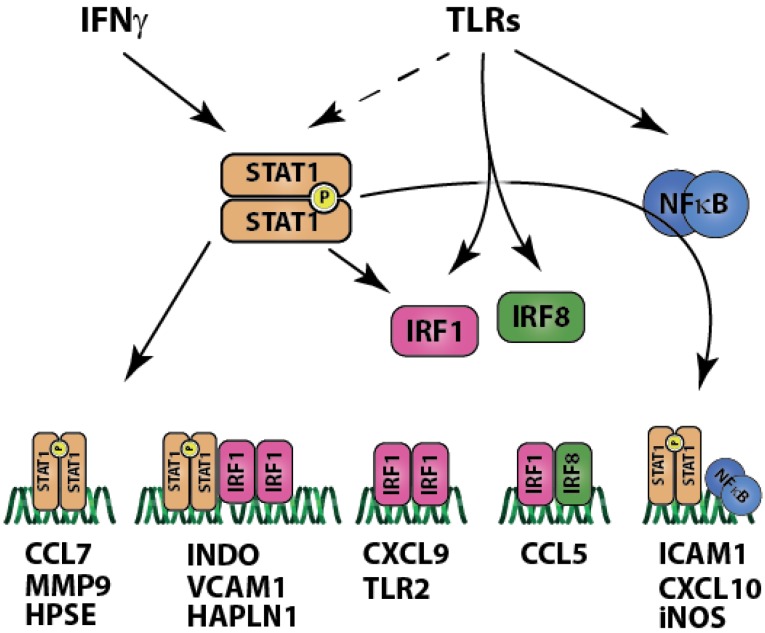
Signal integration between interferon gamma (IFNγ) and Toll-like receptors (TLRs) involves interactions of STAT1, interferon-regulated factor (IRF)1, IRF8 and nuclear factor kappa-light-chain-enhancer of activated B cells (NFκB). Analysis of gene expression profiles of atherosclerotic plaques together with the results of microarray experiments conducted by our group on STAT1 -/- SMC [Chmielewski S. *et al.*] create this complex signal integration picture (see Discussion). STAT1 regulates genes by itself, but also interacting with IRF1, IRF8 or NFκB.

Collectively, these results predict that key processes involved in plaque development are regulated by STAT1, either alone or in close co-operation with NFκB and IRFs providing a platform for cross-talk between different inflammatory stimuli. It also provides further proof for the crucial role of STAT1 in human atherosclerosis. This notion is further supported by an additional study from our group that was recently submitted elsewhere [Chmielewski S. *et al.*], in which we uncovered a subset of 30 STAT1-dependent genes that are highly amplified by co-administration of IFNγ and LPS in VSMCs *in vitro* [Chmielewski S. *et al.*]. These genes included the chemokines CCL5, CXCL10, CCL8, CXCL9, CCRL2, which were also up-regulated in carotid or coronary human plaques, as shown here, and together reflect pro-atherogenic responses in human atherosclerosis. Based on our findings and other studies from the literature, Figure 2 summarizes the complex role of STAT1 in signal integration between IFNγ and TLRs that we propose to take place in the forming atheroma.

Regulatory modules containing elements for STAT1 and IRF or NFκB have been shown to play key role in signal integration of various signaling pathways. For instance, maximal transcription of ICAM1 is possible only when both NFκB and STAT1 are bound to the promoter. The same mechanism was observed for CXCL10 in human smooth muscle cells [44] and monocytic THP-1 cells [45]. We observed a similar synergy between IFNγ and TLR4 signaling in human endothelial cells and murine smooth muscle cells, which was STAT1 dependent and led to amplified expression of CXCL10 and other genes [17] [Chmielewski S. *et al.*]. Furthermore, induction of iNOS expression by LPS is managed by NFκB binding, but presence of a GAS element in the promoter and co-treatment with IFNγ enables maximal expression [46]. Co-operative action of binding sites for STAT1 and IRF1 has been shown to manage expression of indoleamine 2,3-dioxygenase 1 (IDO1) (also termed INDO) gene [21], which is involved in sustaining chronic inflammation and is one of the hallmark genes of the M1 inflammatory macrophage phenotype [47]. Involvement of regulatory modules in complex signal integration of IFNγ and TLR4 (also other TLRs), in addition to other pro-inflammatory signals, has long been appreciated by the scientific community and the evidence presented here suggests that it is a functional mechanism in the plaque development contributing to chronic production of inflammatory modulators.

It is known that the pathophysiology of coronary plaques resembles that of carotid plaques [48]. Prevalence of stenosis in one artery type has been associated with prevalence of stenosis in the other. Both plaque types have been shown to respond in a similar way to shear stress and both display an inflammatory background [49]. We were interested if the degree of similarity between coronary and carotid plaques could be recognized at the gene expression level. Indeed, overlap was observed in up-regulated genes from both datasets, together forming a 72-gene “plaque signature”. As expected, also distinct gene sets could be identified. This was in agreement with the fact that atherosclerosis is a multi-factorial disease, and it is highly likely that factors inducing and accelerating the disease will differ between various plaque types and even between different specimens [50,51,52].

When we characterized in more detail the biological functions of these 72 “plaque signature” genes, we could distinguish many GO terms related to plaque development processes: cell adhesion (ALCAM [53,54], VCAM1 [55]), T-cell migration (CCL5 [56], CCR5 [57], and CXCR4 [58]), response to LPS (TLR4), *etc.* (Table 4 and supplementary data file). In addition, promoter analysis of these genes revealed over-representation of STAT1 binding sites as well as STAT1 containing modules (Table 2). The STAT1-NFκB module was present in 31 genes of this common group while the STAT1-IRF module could be detected in 45 genes. Some of these genes (CCL5, CCL19, CCL4, CXCL10, CXCL2, CXCL9 and MMP9) actually possessed binding sites for STAT1, NFκB as well as IRFs. The idea that STAT1-dependent cross-talk between IFNγ and TLR4 potentially exists in human plaques and the discovery of a “plaque gene signature” present the possibility for development of a novel non-invasive screening assay. Indeed, with respect to the cellular localization of the proteins encoded by the 72 common signature genes many of them are secreted and localize to the extracellular space or cell membrane making them ideal serum markers of atherosclerosis (Figure 1 and Table 5). In addition, the majority has a known connection to atherosclerosis, as determined by literature mining (Figure 1).

## 4. Materials and Methods

### 4.1. Microarray Data Normalization and Analysis

GSE40231 [25], GSE21545 [26], and GSE13760 [59] datasets were downloaded from the National Center for Biotechnology Information (NCBI) Gene Expression Omnibus repository (GEO). We used a microarray dataset obtained from human coronary plaques (GSE40231) published and deposited in NCBI GEO by Hagg *et al.* [25]. The authors acquired 40 coronary plaques from patients, collected during coronary artery bypass surgery and analyzed gene expression profiles using Affymetrix Human Genome U133 Plus2 microarrays. This dataset also included samples obtained from inferior mesenteric arteries (IMA), which were atherosclerosis-free. In addition we analyzed a microarray dataset (GSE21545) obtained from carotid plaques collected during carotid endarterectomy and deposited by Folkersen *et al.* This dataset contained 124 Affymetrix Human Genome U133 Plus2 arrays.

Datasets were normalized in Chipster software using RMA algorithm [60]. Signals were log-transformed and probes were combined to genes using Chipster’s “Combine probes to genes” utility. Healthy arterial tissue microarrays from GSE13760 were used as controls for GSE21545, GSE40231 contained own controls (IMA samples). Batch effects between the combined datasets were removed using ComBat tool, a commonly used method for removing variations between batches of microarrays [61]. Then, log of fold change was calculated for GSE21545 and GSE40231. For further analysis, genes which were statistically significantly up-regulated at least two times were used. The significance was tested with empirical Bayes test adjusted by Bonferroni correction for multiple testing.

### 4.2. Gene Ontology Enrichment Studies

Gene lists were mapped to gene ontology terms from biological_process category and term enrichment was calculated using GOEAST advance tool: Multi-Batch genes [62]. The default settings were used for the analysis: hypergeometric test and Yekutieli multi-test adjustment method. Terms with *p*-values of less than 0.01 were considered significantly enriched. Next, GO terms level 1 and 2 were fed to REVIGO online tool to produce a short list of meaningful non-redundant GO terms. Tables presented in the article contain 20 most enriched terms. Lists of all terms are available in the supplementary data file.

### 4.3. Pathway Analysis and Literature Mining

Pathway analysis and literature mining were done using Genomatix Pathway System [63]. Lists of genes were uploaded to the system and then over-representation of genes related to specific terms based on literature (“literature mining”) or to canonical pathways (“pathway analysis”) was calculated. In case of pathway analysis, the software relies on canonical pathways from databases such as KEGG [64], Biocarta [65] and Reactome [66]. Literature mining uses PubMed database of scientific articles. In both cases, the system calculates a *p*-value, which indicates how strong over-representation of a certain group of genes is in the analyzed dataset. Based on these analyses a Circos plot was generated using the online tool [67].

### 4.4. Promoter Analysis

First, the Genomatix Gene2Promoter tool was used to retrieve human promoters corresponding to gene symbols from the ElDorado database. A total of 1.5 kb upstream and 0.5 kb downstream of the transcription start site was retrieved. The list of STAT1 target genes up-regulated by IFNγ and LPS in murine smooth muscle cells (SMCs) [Chmielewski S. *et al.*, personal communication] was used as the starting point for promoter analysis. That list was fed into the pSCAN online promoter analysis tool in order to look for GAS, ISRE and NFκB binding sites. The software was set to analyze 950 bp upstream and 50 bp downstream of the transcription start site. PSCAN produced a list of over-represented transcription factor binding sites together with occurrences of each site and a matrix similarity score. Occurrences having the score of at least 0.8 were fed into MatDefine (Genomatix software package) to create a highly conserved matrix for each transcription factor binding site. The settings were as follows: touple size—8; no. of sequences containing touple—60%; matrix similarity score for sequence inclusion—0.9. Matrices for GAS, ISRE, and NFκB binding sites were then used in pSCAN as “user supplied matrices” to search for occurrences in genes up-regulated two-fold in atherosclerotic plaque datasets. RegionMiner (Genomatix) was used to assess co-appearance of STAT1 binding sites with either NFκB or IRF binding sites as modules. All presented z-score values were calculated against promoter background. Z-scores are a measure of distance of the tested population from the general population. A positive value means that the number of given transcription factor binding sites is found more often in the tested sample than it would be in a random sample. A negative value means that it is found less often. It is generally accepted to consider z-scores of more than two or less than minus two as significant. It corresponds to a *p*-value of 0.05 [68].

### 4.5. Supplementary Data

The enclosed supplementary data file is available in MS Excel format. It contains several sheets with: two-fold up-regulated genes in carotid and coronary plaques with indicated putative regulatory elements (GAS, ISRE, NFkB, STAT1-NFkB, STAT1-IRF); GO terms significantly enriched in each plaque dataset; a list of common genes between the datasets with indicated link to atherosclerosis (determined by literature mining) and a list of GO terms significantly enriched in the common genes. In addition, gene lists were compared to putative STAT1 targets obtained from a microarray study, in which STAT1-deficient murine vascular smooth muscle cells were treated with IFNγ and/or LPS [Chmielewski S. *et al.*, personal communication]. Genes that came up as STAT1-targets were marked with a “+”. The lists are organized and formatted as tables, which allows for easy filtering of data by using the filter arrows in the heading of each column. 

## 5. Conclusions

We present further proof that IFNγ-TLR cross-talk, which has been shown *in vitro* to exist in many immunomodulatory and vascular cell types, potentially contributes to atherosclerotic plaque development *in vivo*. We are aware of the limitations of our *in silico* study, and realize that detailed promoter mutagenesis or chromatin immunoprecipitation-polymerase chain reaction (ChIP-PCR) experiments on a selection of newly identified STAT1-target genes is necessary to provide final proof for our proposed role of STAT1 in human atherosclerotic plaque development. Nevertheless, it is tempting to speculate that targeted intervention at STAT1 in the vasculature would be beneficial in the management of vascular disease [69]. We have confirmed by data mining that carotid and coronary plaques are indeed very similar. In addition, we observed that many genes involved in plaque development could be regulated in a concerted way by STAT1, NFκB and IRFs. Further analysis of transcription factors activated by other stimuli, such as TGFβ or Angiotensin II, could provide insight into how these pathways interact with the IFNγ-TLR axis.

With the proven role of STAT1-dependent signal amplification in endothelial and vascular smooth muscle cells and also atheroma-interacting immune cells, STAT1 target genes represent promising markers of endothelial dysfunction and atherosclerosis development. Among the 72 genes that were shown to be commonly expressed in carotid and coronary plaques, the majority encoded secreted or membrane bound proteins that could potentially be detected in serum of patients [70]. In addition, many of these genes have known important roles particularly in the early stages of plaque development. Therefore, we propose that a selection of these markers could reflect a specific profile of early plaque formation, allowing for timely atherosclerosis detection, disease progression monitoring, and treatment outcome assessment by a simple blood test. In the clinic, the availability of such an assay would mean non-invasive diagnosis of atherosclerosis and the ability to assess the efficacy of drugs in individual patients. In addition, early diagnosis could possibly improve outcomes and decrease treatment costs.

## Supplementary Files

Supplementary File 1
